# Supplementary material for: Association between different metabolic obesity phenotypes and colorectal adenoma
Source: PLoS One. 2026 Feb 23;21(2):e0343556. doi: 10.1371/journal.pone.0343556 (PMC12928563; doi:10.1371/journal.pone.0343556)
Supplement: S1 Table — Notes: Model 1: not adjusted. Model 2: adjustment for age, and sex. Model 3: adjustment for age, sex, smoking, and drinking. (DOC) [file pone.0343556.s001.doc]

**Table Sensitivity analysis**

|  |  | Model 1 | | Model 2 | | Model 3 | |
| --- | --- | --- | --- | --- | --- | --- | --- |
|  |  | OR (95% CI) | p value | OR (95% CI) | p value | OR (95% CI) | p value |
| **Total** | **n=2042** |  |  |  |  |  |  |
| MHNO | 695 | 1 (Reference) |  | 1 (Reference) |  | 1 (Reference) |  |
| MHO | 177 | 1.206 (0.857, 1.696) | 0.283 | 1.059 (0.737, 1.523) | 0.755 | 1.073 (0.746, 1.545) | 0.704 |
| MUNO | 642 | 1.782 (1.430, 2.221) | <0.001 | 1.301 (1.026, 1.649) | 0.030 | 1.273 (1.003, 1.616) | 0.047 |
| MUO | 528 | 1.918 (1.521, 2.418) | <0.001 | 1.329 (1.034, 1.707) | 0.026 | 1.310 (1.019, 1.684) | 0.035 |

**Notes: Model 1:** not adjusted. **Model 2:** adjustment for age, and sex. **Model 3:** adjustment for age, sex, smoking, and drinking.
