# Supplementary material for: Association between different metabolic obesity phenotypes and colorectal adenoma
Source: PLoS One. 2026 Feb 23;21(2):e0343556. doi: 10.1371/journal.pone.0343556 (PMC12928563; doi:10.1371/journal.pone.0343556)
Supplement: S2 Data — (DOCX) [file pone.0343556.s002.docx]

**Table1 Comparison of Baseline Characteristics of Enrolled Subjects with and without Adenoma**

| **Variables** | **All subjects** | | | **Females** | | | **Males** | | |
| --- | --- | --- | --- | --- | --- | --- | --- | --- | --- |
|  | No Adenoma (n=1166) | Adenoma (n=876) | P-value | No Adenoma (n=686) | Adenoma (n=307) | P-value | No Adenoma (n=480) | Adenoma (n=569) | P-value |
| Age, years | 54.00 [46.00, 61.00] | 59.00 [53.00, 67.00] | <0.001 | 55.00 [47.25, 62.00] | 60.00 [54.00, 67.00] | <0.001 | 53.00 [45.00, 60.00] | 59.00 [53.00, 67.00] | <0.001 |
| Sex(male), n(%) | 480 (41.2) | 569 (65.0) | <0.001 | - | - | - | - | - | - |
| Smoking, n(%) | 189 (16.2) | 257 (29.3) | <0.001 | 8 (1.2) | 4 (1.3) | >0.050 | 181 (37.7) | 253 (44.5) | 0.032 |
| Drinking, n(%) | 188 (16.1) | 244 (27.9) | <0.001 | 28 (4.1) | 10 (3.3) | 0.655 | 160 (33.3) | 234 (41.1) | 0.011 |
| Diabetes, n(%) | 89 (7.6) | 126 (14.4) | <0.001 | 42 (6.1) | 34 (11.1) | 0.010 | 47 (9.8) | 92 (16.2) | 0.003 |
| Hypertention, n(%) | 301 (25.8) | 354 (40.4) | <0.001 | 144 (21.0) | 111 (36.2) | <0.001 | 157 (32.7) | 243 (42.7) | 0.001 |
| BMI,kg/m^2^ | 23.44 [21.34, 25.69] | 24.22 [22.29, 26.23] | <0.001 | 22.48 [20.70, 24.64] | 23.62 [21.66, 25.07] | <0.001 | 24.64 [22.84, 26.96] | 24.57 [22.66, 26.57] | 0.316 |
| SBP,mmHg | 125.00 [115.00, 136.00] | 130.00 [119.00, 141.00] | <0.001 | 123.00 [112.00, 135.00] | 130.00 [119.00, 141.00] | <0.001 | 128.00 [118.75, 137.00] | 130.00 [118.00, 141.00] | 0.021 |
| DBP,mmHg | 79.00 [72.00, 86.00] | 80.00 [72.75, 87.00] | 0.011 | 76.00 [69.00, 83.75] | 78.00 [71.00, 84.00] | 0.056 | 82.50 [75.00, 90.00] | 81.00 [74.00, 89.00] | 0.149 |
| TC,mmol/L | 4.84 [4.23, 5.51] | 4.84 [4.18, 5.44] | 0.197 | 4.92 [4.29, 5.63] | 5.12 [4.49, 5.74] | 0.039 | 4.74 [4.15, 5.39] | 4.70 [4.07, 5.29] | 0.129 |
| TG,mmol/L | 1.31 [0.91, 1.97] | 1.58 [1.07, 2.34] | <0.001 | 1.15 [0.83, 1.65] | 1.51 [1.04, 1.93] | <0.001 | 1.62 [1.09, 2.49] | 1.66 [1.12, 2.57] | 0.770 |
| HDL-C,mmol/L | 1.23 [1.04, 1.48] | 1.17 [0.98, 1.39] | <0.001 | 1.35 [1.16, 1.61] | 1.31 [1.12, 1.52] | 0.010 | 1.09 [0.93, 1.26] | 1.10 [0.93, 1.28] | 0.582 |
| LDL-C,mmol/L | 2.77 [2.24, 3.30] | 2.72 [2.20, 3.22] | 0.167 | 2.83 [2.26, 3.34] | 2.93 [2.32, 3.42] | 0.141 | 2.68 [2.19, 3.19] | 2.63 [2.12, 3.12] | 0.128 |
| FBG,mmol/L | 5.01 [4.68, 5.59] | 5.26 [4.80, 6.12] | <0.001 | 4.97 [4.64, 5.43] | 5.20 [4.76, 5.82] | <0.001 | 5.13 [4.74, 5.79] | 5.29 [4.85, 6.31] | 0.001 |
| Metabolic risk components | 1.00 [0.00, 2.00] | 2.00 [1.00, 3.00] | <0.001 | 1.00 [0.00, 2.00] | 2.00 [1.00, 3.00] | <0.001 | 2.00 [1.00, 3.00] | 2.00 [1.00, 3.00] | 0.001 |

**Table2 Traits of Enrolled Subjects at Baseline in the Different Cohorts of Metabolic Obesity**

| **Variables** | Total  (n=2042) | MHNO  (n=921) | MHO  (n=43) | MUNO  (n=890) | MUO  (n=188) | P-value |
| --- | --- | --- | --- | --- | --- | --- |
| Age, years | 57.00 [49.00, 64.00] | 54.00 [46.00, 61.00] | 55.00 [47.50, 61.00] | 59.00 [53.00, 66.75] | 55.00 [47.00, 61.00] | <0.001 |
| Sex(male), n(%) | 1049 (51.4) | 339 (36.8) | 16 (37.2) | 551 (61.9) | 143 (76.1) | <0.001 |
| Smoking, n(%) | 446 (21.8) | 126 (13.7) | 8 (18.6) | 258 (29.0) | 54 (28.7) | <0.001 |
| Drinking, n(%) | 432 (21.2) | 133 (14.4) | 6 (14.0) | 231 (26.0) | 62 (33.0) | <0.001 |
| Diabetes, n(%) | 215 (10.5) | 12 (1.3) | 0 (0.0) | 169 (19.0) | 34 (18.1) | <0.001 |
| Hypertention, n(%) | 655 (32.1) | 139 (15.1) | 9 (20.9) | 401 (45.1) | 106 (56.4) | <0.001 |
| Adenoma, n(%) | 876 (42.9) | 306 (33.2) | 16 (37.2) | 457 (51.3) | 97 (51.6) | <0.001 |
| BMI,kg/m^2^ | 23.88 [21.72, 25.97] | 22.46 [20.76, 24.22] | 29.38 [28.44, 30.83] | 24.26 [22.53, 25.71] | 29.41 [28.58, 30.51] | <0.001 |
| SBP,mmHg | 127.00 [117.00, 138.00] | 121.00 [112.00, 130.00] | 131.00 [120.00, 142.00] | 132.00 [122.00, 141.00] | 135.00 [123.00, 146.00] | <0.001 |
| DBP,mmHg | 79.00 [72.00, 87.00] | 76.00 [70.00, 83.00] | 81.00 [74.50, 89.00] | 81.00 [74.00, 88.00] | 87.00 [79.00, 93.00] | <0.001 |
| TC,mmol/L | 4.84 [4.21, 5.49] | 4.89 [4.30, 5.49] | 4.88 [4.51, 5.86] | 4.80 [4.11, 5.48] | 4.76 [4.16, 5.39] | 0.033 |
| TG,mmol/L | 1.41 [0.98, 2.13] | 1.06 [0.79, 1.39] | 1.22 [0.93, 1.57] | 1.94 [1.35, 2.75] | 2.20 [1.54, 3.00] | <0.001 |
| HDL-C,mmol/L | 1.20 [1.01, 1.44] | 1.35 [1.18, 1.60] | 1.27 [1.18, 1.45] | 1.07 [0.92, 1.28] | 1.00 [0.87, 1.19] | <0.001 |
| LDL-C,mmol/L | 2.74 [2.22, 3.27] | 2.86 [2.41, 3.30] | 3.04 [2.50, 3.59] | 2.60 [2.05, 3.22] | 2.66 [2.09, 3.19] | <0.001 |
| FBG,mmol/L | 5.11 [4.72, 5.76] | 4.88 [4.60, 5.21] | 4.99 [4.72, 5.28] | 5.58 [4.93, 6.46] | 5.61 [5.00, 6.44] | <0.001 |
| Metabolic risk components | 2.00 [1.00, 3.00] | 1.00 [0.00, 1.00] | 1.00 [1.00, 1.00] | 3.00 [2.00, 3.00] | 3.00 [2.00, 3.00] | <0.001 |

**Table3 Traits of Enrolled Female Subjects at Baseline in the Different Cohorts of Metabolic Obesity**

| **Variables** | Total  (n=993) | MHNO  (n=582) | MHO  (n=27) | MUNO  (n=339) | MUO  (n=45) | P-value |
| --- | --- | --- | --- | --- | --- | --- |
| Age, years | 57.00 [50.00, 63.00] | 54.00 [46.00, 60.00] | 55.00 [49.50, 60.50] | 61.00 [55.00, 68.00] | 61.00 [55.00, 66.00] | <0.001 |
| Smoking, n(%) | 12 (1.2) | 6 (1.0) | 0 (0.0) | 4 (1.2) | 2 (4.4) | 0.218 |
| Drinking, n(%) | 38 (3.8) | 22 (3.8) | 1 (3.7) | 12 (3.5) | 3 (6.7) | 0.785 |
| Diabetes, n(%) | 76 (7.7) | 9 (1.5) | 0 (0.0) | 56 (16.5) | 11 (24.4) | <0.001 |
| Hypertention, n(%) | 255 (25.7) | 79 (13.6) | 6 (22.2) | 143 (42.2) | 27 (60.0) | <0.001 |
| Adenoma, n(%) | 307 (30.9) | 137 (23.5) | 9 (33.3) | 141 (41.6) | 20 (44.4) | <0.001 |
| BMI,kg/m^2^ | 22.83 [20.94, 24.89] | 22.00 [20.40, 23.78] | 29.38 [28.74, 30.67] | 23.71 [21.88, 24.97] | 29.53 [28.58, 30.61] | <0.001 |
| SBP,mmHg | 125.00 [114.00, 137.00] | 119.00 [110.00, 128.00] | 131.00 [120.00, 143.00] | 134.00 [124.00, 143.00] | 133.00 [123.00, 149.00] | <0.001 |
| DBP,mmHg | 77.00 [70.00, 84.00] | 75.00 [68.00, 81.00] | 81.00 [73.50, 87.50] | 80.00 [72.00, 86.00] | 84.00 [77.00, 89.00] | <0.001 |
| TC,mmol/L | 5.00 [4.33, 5.66] | 5.03 [4.38, 5.62] | 5.47 [4.66, 6.22] | 4.94 [4.28, 5.74] | 4.76 [4.25, 5.36] | 0.151 |
| TG,mmol/L | 1.25 [0.88, 1.75] | 1.01 [0.76, 1.34] | 1.29 [1.02, 1.56] | 1.82 [1.28, 2.44] | 1.74 [1.26, 2.56] | <0.001 |
| HDL-C,mmol/L | 1.34 [1.15, 1.57] | 1.44 [1.24, 1.69] | 1.32 [1.19, 1.48] | 1.19 [1.00, 1.40] | 1.20 [1.02, 1.33] | <0.001 |
| LDL-C,mmol/L | 2.86 [2.31, 3.36] | 2.91 [2.40, 3.33] | 3.44 [2.82, 3.76] | 2.69 [2.12, 3.38] | 2.80 [2.07, 3.28] | <0.001 |
| FBG,mmol/L | 5.03 [4.68, 5.59] | 4.84 [4.59, 5.15] | 5.09 [4.84, 5.31] | 5.58 [4.99, 6.32] | 5.65 [5.09, 6.46] | <0.001 |
| Metabolic risk components | 1.00 [0.00, 2.00] | 0.00 [0.00, 1.00] | 1.00 [1.00, 1.00] | 2.00 [2.00, 3.00] | 3.00 [2.00, 3.00] | <0.001 |

**Table4 Traits of Enrolled Male Subjects at Baseline in the Different Cohorts of Metabolic Obesity**

| **Variables** | Total  (n=1049) | MHNO  (n=339) | MHO  (n=16) | MUNO  (n=551) | MUO  (n=143) | P-value |
| --- | --- | --- | --- | --- | --- | --- |
| Age, years | 57.00 [49.00, 64.00] | 56.00 [47.00, 64.00] | 52.50 [41.00, 64.00] | 59.00 [51.00, 66.00] | 53.00 [46.00, 59.00] | <0.001 |
| Smoking, n(%) | 434 (41.4) | 120 (35.4) | 8 (50.0) | 254 (46.1) | 52 (36.4) | 0.007 |
| Drinking, n(%) | 394 (37.6) | 111 (32.7) | 5 (31.2) | 219 (39.7) | 59 (41.3) | 0.134 |
| Diabetes, n(%) | 139 (13.3) | 3 (0.9) | 0 (0.0) | 113 (20.5) | 23 (16.1) | <0.001 |
| Hypertention, n(%) | 400 (38.1) | 60 (17.7) | 3 (18.8) | 258 (46.8) | 79 (55.2) | <0.001 |
| Adenoma, n(%) | 569 (54.2) | 169 (49.9) | 7 (43.8) | 316 (57.4) | 77 (53.8) | 0.139 |
| BMI,kg/m^2^ | 24.61 [22.68, 26.73] | 23.34 [21.53, 24.81] | 29.24 [28.39, 30.65] | 24.61 [23.04, 26.04] | 29.39 [28.57, 30.47] | <0.001 |
| SBP,mmHg | 129.00 [118.00, 139.00] | 124.00 [115.00, 132.00] | 131.50 [121.25, 137.00] | 131.00 [120.50, 140.00] | 136.00 [123.50, 144.50] | <0.001 |
| DBP,mmHg | 82.00 [75.00, 89.00] | 79.00 [72.00, 85.00] | 81.00 [75.00, 89.25] | 82.00 [75.00, 90.00] | 88.00 [81.00, 94.00] | <0.001 |
| TC,mmol/L | 4.72 [4.11, 5.32] | 4.72 [4.20, 5.27] | 4.62 [4.12, 5.09] | 4.71 [3.98, 5.36] | 4.76 [4.14, 5.40] | 0.664 |
| TG,mmol/L | 1.64 [1.10, 2.56] | 1.13 [0.84, 1.46] | 1.16 [0.90, 1.58] | 2.03 [1.40, 2.93] | 2.34 [1.63, 3.22] | <0.001 |
| HDL-C,mmol/L | 1.09 [0.93, 1.27] | 1.23 [1.12, 1.44] | 1.24 [1.15, 1.39] | 1.00 [0.88, 1.19] | 0.96 [0.85, 1.10] | <0.001 |
| LDL-C,mmol/L | 2.67 [2.17, 3.15] | 2.79 [2.41, 3.17] | 2.52 [2.32, 3.01] | 2.54 [2.02, 3.13] | 2.66 [2.12, 3.17] | <0.001 |
| FBG,mmol/L | 5.22 [4.78, 6.06] | 4.93 [4.63, 5.28] | 4.92 [4.58, 5.09] | 5.57 [4.90, 6.52] | 5.55 [4.99, 6.40] | <0.001 |
| Metabolic risk components | 2.00 [1.00, 3.00] | 1.00 [0.00, 1.00] | 1.00 [1.00, 1.00] | 3.00 [2.00, 3.00] | 3.00 [2.00, 4.00] | <0.001 |

**Table5 Results of logistic regression analysis for the relationship between metabolic obesity phenotypes and the occurrence of adenoma based on the sex**

|  | Model 1 | | Model 2 | | Model 3 | |
| --- | --- | --- | --- | --- | --- | --- |
|  | OR (95% CI) | p value | OR (95% CI) | p value | OR (95% CI) | p value |
| **All** |  |  |  |  |  |  |
| MHNO | 1 (Reference) |  | 1 (Reference) |  | 1 (Reference) |  |
| MHO | 1.191 (0.619, 2.219) | 0.589 | 1.226 (0.619, 2.353) | 0.548 | 1.207 (0.608, 2.323) | 0.579 |
| MUNO | 2.121 (1.755, 2.567) | <0.001 | 1.390 (1.130, 1.709) | 0.002 | 1.353 (1.099, 1.666) | 0.004 |
| MUO | 2.142 (1.560, 2.945) | <0.001 | 1.572 (1.122, 2.204) | 0.009 | 1.558 (1.111, 2.187) | 0.010 |
| **Male** |  |  |  |  |  |  |
| MHNO | 1 (Reference) |  | 1 (Reference) |  | 1 (Reference) |  |
| MHO | 0.782 (0.274, 2.147) | 0.634 | 0.873 (0.291, 2.532) | 0.803 | 0.834 (0.276, 2.437) | 0.739 |
| MUNO | 1.353 (1.031, 1.776) | 0.029 | 1.190 (0.895, 1.580) | 0.230 | 1.130 (0.847, 1.505) | 0.405 |
| MUO | 1.174 (0.794, 1.739) | 0.423 | 1.371 (0.911, 2.070) | 0.131 | 1.346 (0.891, 2.041) | 0.159 |
| **Female** |  |  |  |  |  |  |
| MHNO | 1 (Reference) |  | 1 (Reference) |  | 1 (Reference) |  |
| MHO | 1.624 (0.682, 3.611) | 0.248 | 1.548 (0.645, 3.473) | 0.303 | 1.553 (0.647, 3.486) | 0.300 |
| MUNO | 2.313 (1.734, 3.090) | <0.001 | 1.695 (1.244, 2.310) | 0.001 | 1.694 (1.243, 2.308) | 0.001 |
| MUO | 2.599 (1.387, 4.814) | 0.002 | 2.019 (1.061, 3.796) | 0.030 | 2.013 (1.055, 3.797) | 0.031 |

**Notes: Model 1:** not adjusted. **Model 2:** adjustment for age, and sex (only in all participants). **Model 3:** adjustment for age, sex (only in all participants), smoking, and drinking.

**Table6 Results of logistic regression analysis for the relationship between metabolic obesity phenotypes and the occurrence of adenoma based on the age**

|  | Model 1 | | Model 2 | | Model 3 | |
| --- | --- | --- | --- | --- | --- | --- |
|  | OR (95% CI) | p value | OR (95% CI) | p value | OR (95% CI) | p value |
| **All** |  |  |  |  |  |  |
| MHNO | 1 (Reference) |  | 1 (Reference) |  | 1 (Reference) |  |
| MHO | 1.191 (0.619, 2.219) | 0.589 | 1.226 (0.619, 2.353) | 0.548 | 1.207 (0.608, 2.323) | 0.579 |
| MUNO | 2.121 (1.755, 2.567) | <0.001 | 1.390 (1.130, 1.709) | 0.002 | 1.353 (1.099, 1.666) | 0.004 |
| MUO | 2.142 (1.560, 2.945) | <0.001 | 1.572 (1.122, 2.204) | 0.009 | 1.558 (1.111, 2.187) | 0.010 |
| **Age<60 years** |  |  |  |  |  |  |
| MHNO | 1 (Reference) |  | 1 (Reference) |  | 1 (Reference) |  |
| MHO | 1.194 (0.508, 2.600) | 0.666 | 1.158 (0.488, 2.550) | 0.726 | 1.114 (0.467, 2.467) | 0.798 |
| MUNO | 2.207 (1.711, 2.852) | <0.001 | 1.744 (1.330, 2.287) | <0.001 | 1.648 (1.252, 2.169) | <0.001 |
| MUO | 2.137 (1.448, 3.147) | <0.001 | 1.490 (0.986, 2.243) | 0.057 | 1.490 (0.983, 2.253) | 0.059 |
| **Age≥60 years** |  |  |  |  |  |  |
| MHNO | 1 (Reference) |  | 1 (Reference) |  | 1 (Reference) |  |
| MHO | 1.173 (0.392, 3.508) | 0.771 | 1.325 (0.425, 4.113) | 0.621 | 1.337 (0.428, 4.162) | 0.610 |
| MUNO | 1.576 (1.171, 2.126) | 0.003 | 1.438 (1.054, 1.964) | 0.022 | 1.437 (1.052, 1.963) | 0.023 |
| MUO | 2.408 (1.344, 4.444) | 0.004 | 2.325 (1.268, 4.384) | 0.007 | 2.301 (1.252, 4.348) | 0.008 |

**Notes: Model 1:** not adjusted. **Model 2:** adjustment for age (only in all participants), and sex. **Model 3:** adjustment for age (only in all participants), sex, smoking, and drinking.
