# Supplementary material for: Association between different metabolic obesity phenotypes and colorectal adenoma
Source: PLoS One. 2026 Feb 23;21(2):e0343556. doi: 10.1371/journal.pone.0343556 (PMC12928563; doi:10.1371/journal.pone.0343556)
Supplement: Attachment — Submitted filename: Response to Reviewers.docx [file pone.0343556.s004.docx]

Response Letter

Dear Reviewer,

We appreciate the opportunity to revise our manuscript titled “Association between different metabolic obesity phenotypes and colorectal adenoma” and are grateful for the insightful comments provided by the peer-reviewer. Those comments are all valuable and very helpful for revising and improving our paper, as well as the important guiding significance to our researches. We have tried our best to make all the revisions clear, and we hope that the revised manuscript meets the requirements for publication. In the following, we have provided a point-by-point response addressing each issue raised in the peer-review report:

Comment 1: The study used a cross-sectional design, measuring both exposure (metabolic obesity phenotype) and outcome (presence of CRA) at a single time point. This approach cannot establish temporal causality. MHO is known to be a transient state, with many individuals progressing to MUO over time. Since the study only captured a snapshot, it remains unknown how long the MHO individuals had maintained this phenotype or whether they might transition in the future. Thus, the conclusion that "MHO is not associated with CRA risk" may be short-sighted, potentially reflecting insufficient exposure duration rather than a true lack of association. A prospective cohort study would be the ideal design to address this, though this remains an irreparable limitation of the current study. This point should be discussed more thoroughly in the manuscript.

Response: We agree with the reviewer that the cross-sectional design is a limitation for establishing causality and that the transient nature of the MHO phenotype is an important consideration. As suggested, we have now added a detailed discussion on this limitation in the Discussion section of the manuscript(Line 428 to Line 439).

Comment 2: There is no universally accepted definition for "metabolically healthy" and "metabolically unhealthy" (e.g., some use ATP-III metabolic syndrome criteria, others use HOMA-IR for insulin resistance). Varying definitions can significantly alter the classification of MHO/MUNO individuals, directly affecting the stability and comparability of the results. The manuscript must transparently report the specific criteria used (including BMI cutoffs, metabolic indicators, and their thresholds). Where possible, sensitivity analyses using multiple established definitions should be conducted to test the robustness of the findings.

Response: We sincerely thank the reviewer for this critical and constructive comment. We agree that the lack of a universal definition is a key consideration. We have now explicitly and transparently detailed the specific criteria used to define metabolic health status and obesity in the Methods section(Line 121 to Line 132). This includes the exact components, cut-off values, and the reference standards we adopted; While we did not measure leptin or fasting insulin levels, which precluded a sensitivity analysis using HOMA-IR, we fully recognized the importance of testing the robustness of our findings. Therefore, we conducted an alternative sensitivity analyses using the data available to us: We altered the definition of obesity. In addition to the primary definition of BMI ≥ 28 kg/m² (Chinese criteria), we tested the World Health Organization (WHO) standard for Asian populations (BMI ≥ 25 kg/m²) to define obesity(Line 291 to Line 296).

We are pleased to report that the results of both sensitivity analyses were consistent with our primary findings. The core conclusion—that metabolically unhealthy phenotypes, irrespective of obesity status, are associated with higher odds of CRA—remained robust. These new results have been added to the Results sections of the revised manuscript (see Supplementary Tables S1).

Comment 3: In the male subgroup, no significant differences were observed among the four phenotypes, contradicting the results in the overall and female populations, may be due to: (a) a genuine biological effect—metabolic abnormalities may influence CRA risk differently in men (e.g., via interaction with sex hormones); or (b) insufficient sample size—particularly in the MHO and MUO male subgroups, leading to low statistical power and an inability to detect actual differences (i.e., a false negative). Therefore, it is crucial to report the exact sample sizes (n) for each subgroup. If samples are small, the authors should explicitly state that the negative results may be due to low power and interpret them cautiously. Larger studies are needed to validate these sex-specific differences.

Response: We agree that the sample size in subgroups is a key issue. We have now: First, clearly reported the exact sample sizes (n) for each phenotype within the male and female subgroups in the corresponding Figures(Figure 2 and 3). Second, added a statement in the Results and Discussion sections to caution the interpretation of the non-significant finding in the male subgroup, explicitly acknowledging the possibility of a false negative due to limited sample size and lower statistical power(Line 394 to Line 400).

Comment 4: The study conducted numerous statistical comparisons (across four phenotypes in the overall population, males, females, and age groups <60/≥60 years), but did not mention whether correction for multiple testing (e.g., Bonferroni correction) was applied. When performing extensive subgroup analyses, a stricter significance level (e.g., p < 0.01) or appropriate multiplicity adjustment should be used, and uncorrected results must be interpreted with caution.

Response: We sincerely thank the reviewer for raising this crucial methodological point. We agree that appropriate control for multiple testing is essential. Therefore, in the revised manuscript, the primary analysis in the overall population, which tests our main hypothesis, is interpreted at the conventional significance level of P < 0.05. For the extensive subgroup analyses (by sex and age), we applied a more stringent significance level of P < 0.01 for all tests within these subgroups, as detailed in the 'Statistical Analysis' section of the Methods(Line 151 to Line 155). This approach balances the need to mitigate the inflation of Type I error while maintaining reasonable power to detect potential effects in these secondary analyses.

We have explicitly stated this strategy in the methods, noted it in the results (including table footnotes) and discussed the cautious interpretation of subgroup findings in the context of multiple testing in the Discussion section(Line 394 to Line 400). We believe this significantly strengthens the statistical rigor and interpretation of our findings.

We appreciate your time and consideration, and we anticipate your favorable response at your earliest convenience.

Sincerely yours,

Dr. Qin Zhu

Professor

Department of Gastroenterology,

Zhejiang Hospital

HangZhou 310013, P. R. China

Tel: (86)-17706413537

Email: zhuqin-1@163.com
