# Supplementary material for: Association between different metabolic obesity phenotypes and colorectal adenoma
Source: PLoS One. 2026 Feb 23;21(2):e0343556. doi: 10.1371/journal.pone.0343556 (PMC12928563; doi:10.1371/journal.pone.0343556)
Supplement: Attachment — Submitted filename: Response_to_Reviewers_auresp_2.docx [file pone.0343556.s005.docx]

Response Letter

Dear Editor and Reviewers,

Thank you very much for your comments regarding our manuscript entitled “Association between different metabolic obesity phenotypes and colorectal adenoma” We are truly grateful to your valuable comments and thoughtful suggestions on how to improve our manuscript. The valuable comments have provided important guidance for our research and the revision of our manuscript. Based on these comments and suggestions, we have made appropriate modifications on the original manuscript, we hope that the revised manuscript meets the requirements for publication. In the following, we have provided a point-by-point response addressing each issue raised by the academic editor and reviewer:

**Responds to academic editor’ comments:**

**Comment 1:** Emphasize more clearly in the Discussion that the cross-sectional design does not allow causal inference.

**Response:** We agree with the editor that the cross-sectional design is a limitation for establishing causality. As suggested, we have now explicitly stated that due to the cross-sectional nature of our study, the observed associations cannot be interpreted as causal relationships in the Limitation section of the manuscript(Line 462 to Line 464).

**Comment 2:** Note that the metabolically healthy obesity (MHO) phenotype may be transient and that long-term cohort studies are needed to evaluate phenotype transitions and CRA risk.

**Response:** We sincerely thank the editor for this critical and constructive comment. We added a paragraph in the Discussion section noting that the MHO phenotype is potentially dynamic and may transition to metabolically unhealthy states over time. We now emphasize that prospective cohort studies are required to track these transitions and their longitudinal relationship with CRA risk(Line 413 to Line 417). And in the Limitation section, we also noted that this study have conclusion bias due to its short observation period, and emphasized the need for future long-term prospective research(Line 471 to Line 473).

**Comment 3:** Acknowledge the limitation of defining obesity using BMI alone, and highlight the absence of central obesity indicators (e.g., waist circumference, body fat percentage). Indicate that future studies should integrate body composition measures.

**Response:** We agree that defining obesity using BMI alone is a key issue. We expanded the Limitations subsection to acknowledge that using BMI as the sole criterion for obesity is a limitation, as it does not capture fat distribution or body composition. We specifically mention the lack of central obesity measures (e.g., waist circumference) and body fat percentage data. We have added a sentence suggesting that future studies should incorporate these measures to provide a more comprehensive assessment(Line 474 to Line 478).

**Comment 4:** Clearly report subgroup sample sizes (especially in male phenotypes) in the Results tables.

**Response:** We agree that clearly report subgroup sample sizes is essential. Therefore, in the revised manuscript, The sample sizes for all subgroups (with particular attention to male phenotype categories) have been explicitly specified by adding a separate row in the relevant results tables (Tables 2 and 3).

**Comment 5:** In the Discussion, interpret non-significant findings in males cautiously and acknowledge the possibility of limited statistical power and potential false-negative results.

**Response:** In the Discussion section, when interpreting the results for males, we now explicitly state that the non-significant findings should be interpreted with caution. We acknowledge that these analyses, particularly within certain phenotype strata, may have been underpowered, increasing the possibility of false-negative results(Line 431 to Line 434).

**Comment 6:** Expand the limitations to mention unmeasured lifestyle confounders such as diet and physical activity, and discuss their potential impact on the observed associations.

**Response:** We agree with the editor that we did not fully control for confounding factors.We have expanded the limitations section of the discussion as suggested. We acknowledge that unmeasured or inadequately measured potential confounders, such as dietary patterns and physical activity, may have influenced the findings presented in this paper(Line 478 to Line 482).

**Comment 7:** Strengthen the mechanism section by briefly discussing metabolic features that may be particularly relevant in the Chinese population, supported by existing literature.

**Response:** We agree it is necessary to briefly discuss the mechanisms of metabolic profile intensification that are particularly relevant to the Chinese population. We strengthened the discussion at the mechanistic level by integrating research evidence from studies conducted in Chinese and East Asian populations. We briefly explore population-specific metabolic and dietary characteristics, such as the tendency toward visceral fat accumulation at lower BMI thresholds and a predominantly high-carbohydrate diet, and how these factors differentially influence obesity tolerance phenotypes and their association with colorectal adenoma risk(Line 383 to Line 403). To support this context-specific discussion, we have supplemented relevant literature citations(Line 630 to Line 648).

**Comment 8:** Add a short comparison between your definition of metabolic health and other commonly used definitions.

**Response:** We agree with the editor for the useful suggestion.We have added a section to our discussion briefly comparing our definition with other methods (which may include waist circumference and insulin resistance). Our definition is both suitable for comparison with a large-scale of existing literature and clinically practical(Line 311 to Line 319).

**Comment 9:** Justify the rationale for selecting the current classification to enhance interpretability and comparability with other studies.

**Response:** We clearly justify our choice by stating that this definition was selected to balance clinical relevance with the available data in our study, and to enhance comparability with a significant body of existing literature that uses similar criteria, thereby facilitating the interpretation of our findings within the broader research context.(Line 311 to Line 319).

**Responds to reviewer’s comments:**

**Comment 1:** The cross-sectional design cannot establish a temporal causal relationship between metabolic obesity phenotypes and CRA. Additionally, the MHO phenotype is transient, and the existing conclusion that "MHO is not associated with CRA risk" may be limited by the duration of phenotype observation. It is recommended to further clarify in the discussion that prospective cohort studies are needed in the future to track the long-term impact of phenotype transition on CRA occurrence, avoiding conclusion bias caused by short-term observation.

**Response:**We fully agree with this important point. In the revised Discussion section, we have:

Clearly stated in a dedicated limitations paragraph that due to the cross-sectional design, our findings represent associations and cannot be interpreted as evidence of a temporal or causal relationship between metabolic phenotypes and CRA(Line 462 to Line 464).

Added a specific discussion on the dynamic nature of the MHO phenotype. We now explicitly note that as our study captured metabolic health at a single time point, we cannot account for potential transitions from MHO to metabolically unhealthy states over time. We have revised the text to clarify that our conclusion regarding MHO and CRA risk is necessarily constrained by this snapshot assessment. Furthermore, we strongly emphasize that future longitudinal cohort studies are essential to track these phenotype transitions and to definitively evaluate their long-term impact on CRA development, thereby avoiding bias from short-term observation. (Line 413 to Line 417).

**Comment 2:** Obesity is only defined by BMI, without including complementary indicators reflecting central obesity such as waist circumference and body fat percentage. However, central obesity is more closely associated with metabolic abnormalities and intestinal lesions. It is suggested to supplement the discussion on this limitation and point out that future studies should integrate body composition analysis to refine the impact of different obesity subtypes.

**Response:** We thank the reviewer for highlighting this key limitation. We have expanded the Limitations subsection in the Discussion to explicitly acknowledge that using BMI as the sole measure is a significant constraint. We note that BMI does not distinguish fat distribution or differentiate between fat and lean mass. We specifically mention the absence of central obesity indicators like waist circumference or body fat percentage, which are more strongly correlated with visceral adiposity, metabolic dysfunction, and, as the reviewer notes, intestinal pathology. To address this, we have added a sentence recommending that future research should integrate detailed body composition analysis to refine the understanding of how different obesity subtypes influence CRA risk(Line 474 to Line 478).

**Comment 3:** The sample sizes of some phenotypes in the male subgroup are relatively small, leading to insufficient statistical power which may mask true associations. It is recommended to clearly mark the sample size of each subgroup in the result tables, and emphasize in the discussion that the "no significant difference in males" should be interpreted with caution. The false negative result cannot be excluded due to limited sample size, and larger-scale studies are required to verify the biological mechanisms underlying sex differences.

**Response:** We appreciate the reviewer's careful attention to this detail. We have taken the following actions:

The sample size (n) for each phenotype subgroup within the male and female cohorts is now clearly presented in the separate row in the relevant results tables (Tables 2 and 3).

In the Discussion section, when interpreting the sex-specific results, we have added a clear cautionary statement. We now explicitly state that the lack of statistically significant associations observed among males should be interpreted with caution due to the relatively smaller sample sizes within some phenotype strata, which may have resulted in limited statistical power. We acknowledge that this increases the possibility of Type II error (false-negative results) and that larger-scale studies are required to confirm or refute these findings and to robustly explore the biological mechanisms underlying the apparent sex differences(Line 431 to Line 434).

**Comment 4:** The current confounding factors only include smoking and alcohol consumption, without incorporating key lifestyle factors such as dietary structure and physical activity, which may simultaneously affect metabolic status and CRA occurrence. It is recommended to supplement the discussion on the potential impact of this omission on the results or explain it in the limitations section.

**Response:** This is a valid and important point. We have added a statement to the Limitations paragraph of the Discussion. We now explicitly state that although we adjusted for several important factors, we did not have comprehensive data on other potential confounders, most notably detailed dietary habits and objective measures of physical activity. We acknowledge that unmeasured or inadequately measured potential confounders, such as dietary patterns and physical activity, may have influenced the findings presented in this paper(Line 478 to Line 482).

**Comment 5:** The mechanism section only generally describes the role of hyperglycemia and dyslipidemia, lacking targeted analysis of the metabolic characteristics of the Chinese population. It is suggested to briefly supplement the specificity of relevant mechanisms in the Chinese population by referencing existing literature to enhance the depth of the discussion.

**Respond：**We thank the reviewer for this suggestion to enhance the depth of our discussion. In the revised Mechanism section of the Discussion, we have incorporated a brief, targeted discussion supported by relevant literature. We strengthened the discussion at the mechanistic level by integrating research evidence from studies conducted in Chinese and East Asian populations. We briefly explore population-specific metabolic and dietary characteristics, such as the tendency toward visceral fat accumulation at lower BMI thresholds and a predominantly high-carbohydrate diet, and how these factors differentially influence obesity tolerance phenotypes and their association with colorectal adenoma risk(Line 383 to Line 403). To support this context-specific discussion, we have supplemented relevant literature citations(Line 630 to Line 648).

**Comment 6:** Supplementary Explanations for Definitions and Classifications. Although the definition standards of metabolic health have been clarified, there is no universal standard for "metabolic health". It is recommended to compare the definition used in this study with other mainstream definitions in the discussion, explain the rationality of the selected standard, and enhance the comparability of results.

**Respond：**We have addressed this point by adding a new paragraph in the discussion section. In this paragraph: We briefly compare our definition with other commonly used criteria in the literature (which may include waist circumference and insulin resistance).We justify our selection by explaining that this definition was chosen for its clinical practicality, alignment with several major epidemiological studies, and suitability for our available data. We state that this approach was taken to enhance the interpretability and comparability of our results with a substantial body of existing research(Line 311 to Line 319).

We appreciate your time and consideration, and we anticipate your favorable response at your earliest convenience.

Sincerely yours,

Dr. Qin Zhu

Professor

Department of Gastroenterology,

Zhejiang Hospital

HangZhou 310013, P. R. China

Tel: (86)-17706413537

Email: zhuqin-1@163.com
